# RETRACTED: Qureshi et al. Synthesis and Evaluation of Anti-HIV Activity of Mono- and Di-Substituted Phosphonamidate Conjugates of Tenofovir. *Molecules* 2022, *27*, 4447

**DOI:** 10.3390/molecules30163414

**Published:** 2025-08-19

**Authors:** Aaminat Qureshi, Louise A. Ouattara, Naglaa Salem El-Sayed, Amita Verma, Gustavo F. Doncel, Muhammad Iqbal Choudhary, Hina Siddiqui, Keykavous Parang

**Affiliations:** 1 H.E.J. Research Institute of Chemistry, International Center for Chemical and Biological Sciences, University of Karachi, Karachi 75270, Pakistan; 2 CONRAD, Department of Obstetrics and Gynecology, Eastern Virginia Medical School, Norfolk, VA 23507, USA; yaoal@evms.edu (L.A.O.);; 3 Center for Targeted Drug Delivery, Department of Biomedical and Pharmaceutical Sciences, Chapman University School of Pharmacy, Harry and Diane Rinker Health Science Campus, Irvine, CA 92618, USAamita.verma@shiats.edu.in (A.V.); 4 Cellulose and Paper Department, National Research Center, Dokki, Cairo 12622, Egypt; 5 Bioorganic and Medicinal Chemistry Research Laboratory, Department of Pharmaceutical Sciences, Sam Higginbottom University of Agriculture, Technology and Sciences, Prayagraj 211007, India; 6 Dr. Panjwani Center for Molecular Medicine and Drug Research, International Center for Chemical and Biological Sciences, University of Karachi, Karachi 75270, Pakistan; 7 Department of Biochemistry, King Abdul Aziz University, Jeddah 21452, Saudi Arabia; 8 Department of Chemistry, Faculty of Science and Technology, Universitas Airlangga, Komplek Campus C, Surabaya 60115, Indonesia

The journal retracts the article entitled “Synthesis and Evaluation of Anti-HIV Activity of Mono- and Di-Substituted Phosphonamidate Conjugates of Tenofovir” [1], cited above.

Following publication, the corresponding authors, on behalf of all the authors, brought concerns to the attention of the publisher regarding the validity of the NMR and mass spectra data presented in this article [1]. These concerns were brought to the Editorial Office voluntarily in the interest of upholding the integrity of the scientific record.

Adhering to our standard procedure, an investigation was conducted by the Editorial Office and Editorial Board, with input provided by an internal institutional investigation by H.E.J. Research Institute, conducted at the request of the corresponding author. During the follow-up process, the Editorial Board invited the authors to directly address the concerns and prepare a potential post-publication amendment to the article. Following a review of this proposal, the Editorial Boad determined that this proposal could not sufficiently address the concerns without a significant impact on the study’s central findings. As a result, the Editorial Board have decided to retract this article [1] as per MDPI’s retraction policy (https://www.mdpi.com/ethics#_bookmark30, accessed on 10 July 2025). The authors are invited to present revised findings in a new manuscript. 

This retraction was approved by the authors and by the Editor-in-Chief of *Molecules*.

Louise A. Ouattara, Naglaa Salem El-Sayed, Amita Verma, Gustavo F. Doncel, Muhammad Iqbal Choudhary, Hina Siddiqui, and Keykavous Parang agreed to this retraction. The remaining author did not provide a comment on this decision.
